# Twentieth Century Practice

**Published:** 1898-01

**Authors:** 


TWENTIETH CENTURY PRACTICE.—An International Encyclopedia of Modern Medical Science. By leading authorities of Europe and America. Edited by Thomas L. Stedman, M. D., New York City. In twenty volumes. Volume XII, Mental Diseases, Childhood and Old Age. William Wood & Company, Publishers, 43, 45, 47 East Tenth Street, New York City.

The list of contributors to this volume are all foreigners. The opening article is by G. Fielding Blandford, M. D., F. R. C. P., Lecturer on Psychological Medicine at St. George's Hospital, London, on the subject of insanity. This article covers about 250 pages of the book, and it is a valuable contribution to the subject. Another important contribution is the article on Idiocy, by Paul Sollier, M. D., Professor of Hygiene, Training Schools for Nurses of Paris; Chief of Clinic Adjunct for Mental Diseases, University of Paris.

Criminal Anthropology is the subject of a very interesting paper by Cesare Lombroso, M. D., Professor of Legal Medicine and Psychiatry, University of Turin. The discussion of this covers about fifty pages, and embraces the subheadings of crime in nature, pathological anatomy of the criminal, anomalies in the living, mental characteristics, criminal jargon, relapses of criminals, varieties of criminals, etiology of crime, therapeutics of crime. In treating of the etiology of crime, the author mentions particularly atmospheric influences, the influence of race, of civilazation and barbarism, of food, alcohol, heredity, age, sex, occupation and education.

Under the title "Old Age," J. Boy-Teissier, M. D., Physician to the Hospital Sainte-Marguerite, Marseilles, discusses normal senility; means of combating senility, death from old age, and ordinary diseases in the senile.

The last part of the volume embracing some 300 pages, is devoted to the consideration of Diseases of Children (excluding infectious diseases and rachitis), and was written by Jules Comby, M. D., Physician-in-Chief to the Hospital Trousseau, and editor of *La Medicine Infantile*, Paris. This is a concise presentation of the diseases of children, their etiology, pathology, symptoms, diagnosis and treatment. It is ably presented and thoroughly practical. Its conciseness is the more noticeable because of the very exhaustive discussion of all other subjects which have preceded it in this valuable work.

S. E. H.

---

**The Ladies' Home Journal** for 1898, will publish, among other good things, "The Inner Experiences of a Cabinet Member's Wife." The biographies of President McKinley, Mrs. Cleveland, Mark Twain, Thomas Edison, and Joseph Jefferson will be presented in a novel way by a series of anecdotes, giving the vital characteristics of each. Rev. John Watson, D. D. ("Ian Maclaren"), will contribute a series of articles on matters close to the interest of every man and woman; Edward W. Bok will have a special page for young men, in addition to his usual editorial discussions; Lilian Bell will continue her bright, crisp letters from European capitals; Mrs. Burton Harrison will describe society at the beginning of the century, and ex-President Harrison is to write on "The Flag in the Home."

Two fiction issues, in all over thirty short stories, are promised during the year. The stories will be by Mark Twain, F. Marion Crawford, Hamlin Garland, Mary E. Wilkins, Julia Magruder, Clara Morris, Mrs. A. D. T. Whitney and other well known authors.

The musical announcements for next year include Sousa's newest composition, "The Lady of the White House," dedicated by special permission to Mrs. McKinley; sacred songs and hymns by Fanny Crosby, the blind hymn-writer; Ira D. Sankey, and others quite as prominent in their respective fields.

"Inside of a Hundred Homes" will be continued and supplemented by other articles upon fitting, furnishing and beautifying the home; and in addition to the *Journal's* "Moderate-Cost

Houses," churches, schools, farm buildings, etc., will be given—with detailed plans and specifications.

Mrs. S. T. Rorer, it is announced, will continue to write exclusively for the *Journal*. In addition to her "Cooking Lessons" she will write of foods, their value and their healthfulness.

By the Curtis Publishing Company, Philadelphia. Ten cents per copy; one dollar per year.

